# Device measured physical activity among women with and without adverse pregnancy outcomes: Data from the HUNT study

**DOI:** 10.1371/journal.pone.0357050

**Published:** 2026-09-08

**Authors:** Signe Nilssen Stafne, Vegar Rangul, Ingrid Aanesland Dahle, Laura Vatn Slapgaard, Julie Horn

**Affiliations:** 1 Department of Clinical and Molecular Medicine, Norwegian University of Science and Technology, Trondheim, Norway; 2 Clinic of Rehabilitation, St. Olavs University Hospital, Trondheim, Norway; 3 HUNT Research Centre, Department of Public Health and Nursing, Norwegian University of Science and Technology, Trondheim, Norway; 4 Levanger Hospital, Nord-Trøndelag Hospital Trust, Levanger, Norway; 5 Department of Obstetrics and Gynecology, Levanger Hospital, Nord-Trøndelag Hospital Trust, Levanger, Norway; Hospital Sant Joan de Déu, SPAIN

## Abstract

**Background:**

Women with a history of adverse pregnancy outcomes (APOs), including gestational diabetes mellitus (GDM), hypertensive disorders of pregnancy (HDP), small for gestational age (SGA) offspring, preterm birth and placental abruption, are at increased risk of cardiovascular disease (CVD) and diabetes mellitus (DM) later in life. Physical inactivity is a risk factor for developing CVD and DM. Thus, for women with a history of APOs, it is particularly important to achieve recommended levels of physical activity (PA). The aim of this study was to investigate differences in PA levels among women with and without a history of APOs, using accelerometers to objectively measure PA levels.

**Methods:**

This is a population-based cohort study. Women who participated in the fourth survey of Trøndelag health study (HUNT4) and were registered with one or more births in the Medical Birth Registry of Norway (MBRN) before participation, were included in the study. PA levels were objectively measured with two accelerometers worn by the participants for 3–7 consecutive days. Three different paces of walking, running and cycling were measured by the accelerometers and used to calculate our two outcomes: moderate-vigorous physical activity (MVPA) in metabolic equivalent of task (MET)-minutes/week and total PA in min/day. MVPA included moderate and fast walking, running and cycling, and total PA included additionally slow walking. We used linear and logistic regression to examine the associations between history of APOs and PA levels.

**Results:**

Among 3771 women, 868 (23.0%) had a history of one or more APOs. We found that women with a history of APOs had lower levels of PA. These differences diminished after accounting for postpartum general health and modifiable lifestyle factors. Among women with and without a history of APOs, respectively 17.7% and 15.9% did not achieve recommended levels of PA.

**Conclusions:**

There were no significant associations between history of APOs and PA levels in the fully adjusted regression analyses. Nevertheless, almost every fifth woman (15.9–17.7%) did not achieve recommended levels of PA, and postpartum follow up of women with APOs aimed at increasing their PA is important to prevent CVD and DM later in life.

## Introduction

A pregnancy is considered as a comprehensive, time-limited acute physiological stress test that challenges cardiovascular and metabolic systems. Maladaptive maternal vascular, endocrine and metabolic responses may manifest as adverse pregnancy outcomes (APOs) [1]. Multiple studies have shown that APOs can unmask or exacerbate pre-existing health issues, leading to an accelerated risk of diabetes mellitus (DM) and cardiovascular disease (CVD) later in life [2–5]. Thus, APOs can be used as a predictive window into a woman’s future health.

Physical activity (PA) provides numerous health benefits and significantly reduces the risk of premature morbidity and mortality in a dose–response relationship [6]. It plays an important role in both primary and secondary prevention of several chronic medical conditions, such as DM and CVD [7]. PA is especially beneficial for women with APOs. It improves insulin sensitivity and glucose regulation, helps control blood pressure, supports endothelian function, enhances lipid profiles, and reduces inflammation. These effects can lower the elevated risk of future DM and CVD [8]. A healthy lifestyle that includes regular PA is therefore strongly recommended after APOs [9,10].

The WHO recommends that adults, including pregnant and postpartum women, engage in at least 150–300 minutes of moderate-intensity or 75–150 minutes of vigorous-intensity physical activity per week [11]. This is equivalent to 500–1000 metabolic equivalent of task (MET) minutes of moderate-vigorous physical activity (MVPA) per week. However, few adults meet these recommendations, and women are less active than men globally [12]. Research on lifestyle behaviors in postpartum women is limited and suggests that women with a history of APOs do not consistently engage in more health-promoting behaviors compared to women without such a history [13–20]. Most previous studies have relied on self-reported PA, which is less reliable due to recall and reporting bias [21].

The primary aim of this study is to compare device-measured physical activity (PA) levels during the postpartum period between women with and without a history of adverse pregnancy outcomes (APOs). By employing accelerometry rather than self-report, the study addresses a critical gap in objective surveillance of PA in this high-risk population. Generating robust empirical evidence on PA patterns among women with prior APOs will inform the development of targeted postpartum interventions, with the overarching goal of improving maternal health and mitigating long-term risk.

## Materials and methods

### Study population

This is a population-based cohort study utilizing data from the fourth survey of the Trøndelag Health Study (HUNT4), linked to information on pregnancy outcomes from the Medical Birth Registry of Norway (MBRN), using the unique 11-digit national identity number of Norwegian citizens. The HUNT study is a large population-based cohort study, which invites all adult residents of the northern part of Trøndelag county to participate in extensive health surveys approximately every decade. Information on participants’ health and lifestyle factors were collected through questionnaires, interviews, clinical measurements and biological sampling. The fourth survey, HUNT4, was conducted in 2017–2019 and included, for the first time, objective measurement of PA [22]. The participation rate was 58.8% among women [22]. The MBRN is a mandatory national registry that was initiated in 1967. It collects information on all births in Norway, including complications during pregnancy and delivery outcomes of interest in this study. Data was accessed for research purposes 16^th^ of August 2024. The authors had no access to information that could identify individual participants during or after data collection.

**Fig 1:**
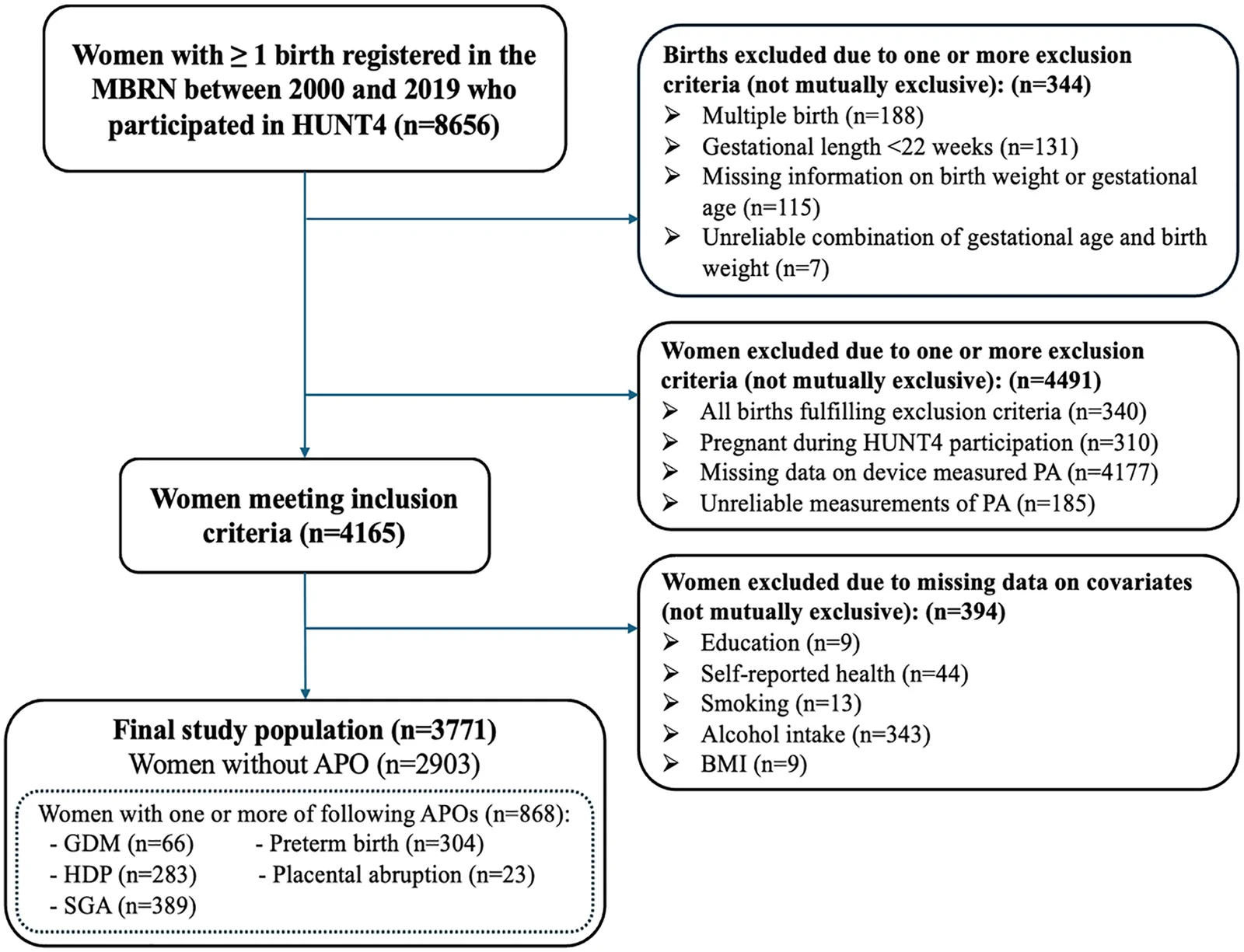
Flowchart of the study population.

The study population includes 8,656 female HUNT4 participants, 20 years or older, who had one or more births recorded in the MBRN between 2000 and 2019 (Fig 1). We chose this time frame since we were interested in PA levels among women with minor children. We excluded births that were multiples, had a gestational length <22 weeks, had missing data on birth weight or gestational length or had an unreliable combination of birth weight and gestational length. A total of 340 women had only births fulfilling one or several exclusion criteria and were excluded from the analyses. We also excluded women who were pregnant during HUNT4 participation (n = 310) since pregnancy may affect PA levels, women with missing data on PA measurements (n = 4177), and women with unreliable measurements of PA (n = 185). Unreliable measurements of PA were defined as one day or more with less than 3 minutes of walking/day. Finally, we excluded women with incomplete data on included covariates (n = 394). The final study population comprised 3771 women.

This study was reported according to the Strengthening the Reporting of Observational Studies in Epidemiology (STROBE) reporting guidelines.

### Outcome measure: physical activity

Physical activity (PA) was objectively measured using two AX3 tri-axial accelerometers (Axivity, Newcastle, UK). The AX3-sensors are small, waterproof devices that were placed on the right thigh and on the lower back. The participants were instructed to wear the accelerometers for seven consecutive days. After ended period of measurement, the participants delivered the devices at the clinical examination site or sent them back in pre-stamped envelopes. To predict the different measurements of interest; walking, running and cycling, a trained machine learning model (eXtreme Gradient Boosting (XGBoost)) was used [23]. In our analyses, all women with a least three valid days of accelerometer recordings were included.

The primary outcomes were Moderate-to-vigorous physical activity (MVPA), expressed as MET minutes per week, and total physical activity, expressed as minutes per day of total PA. MET is a measurement that expresses the intensity of different activities (mL O_2_/kg/min). The energy expended by an individual while seated at rest is equivalent to 1 MET (3.5 mL O_2_/kg/min) [24]. MVPA is widely recognized as a standard for assessing exercise intensity [11], while total PA reflects everyday activity. This provides a comprehensive overview of PA levels in the population.

To calculate MVPA in MET-min/week, each activity was weighted by their MET value according to intensity of the activity [24]. Slow walking was defined as ≤4 km/h, moderate walking as 4.1–5.4 km/h and fast walking as ≥5.5 km/h. Slow walking was weighted 2.8 METs, moderate walking 3.5 METs, fast walking 4.5 METs, running 6.5 METs and cycling 5.8 METs. The total minutes of each activity were added up and divided by the number of days each participants used the accelerometers. The average daily minutes were then multiplied by seven and by the weighted MET value. Eventually, moderate walking, fast walking, running and cycling were added up to calculate the total MVPA in MET-min/week. For total PA we included slow, moderate and fast walking, running and cycling. The total minutes of each activity were added and divided by the number of days the woman used the accelerometers.

We also categorized the two outcomes into three groups each. The international recommendations for MVPA are minimum 500–1000 MET-min/week [11]. MVPA was therefore categorized in <500 MET-min/week, 500–1000 MET-min/week and >1000 MET-min/week to be able to assess the association between history of APOs and minimum recommended, respective higher level of MVPA. We categorized total PA in tertials; < 33^rd^ percentile (<98 min/day), 33^rd^ to 66^th^ percentile (98–126 min/day) and >66^th^ percentile (>126 min/day), to calculate the odds of being less or more active than the middle percentile group.

### Exposure variables: Adverse pregnancy outcomes

Exposure was defined as a history of one or more of the following APOs; gestational diabetes mellitus (GDM), hypertensive disorders of pregnancy (HDP), small for gestational age (SGA) offspring, preterm birth, and placental abruption. The MBRN provided information on APOs and women registered with APOs in one or more pregnancies were considered as exposed.

In the MBRN, diagnoses of APOs were made according to current nationally recommended guidelines. From 2000–2017, GDM was defined by the WHO 1999 criteria. These criteria include fasting plasma glucose ≥7.0 mmol/l or glucose levels ≥7.8 mmol/l two hours after 75g oral glucose tolerance test (OGTT). In 2017, a new national guideline was published, and the criteria changed to fasting plasma glucose level of 5.3–6.9 mmol/L and/or a plasma glucose level of 9.0–11.0 mmol/L after OGTT [25]. A history of HDP included gestational hypertension (GH) and/or preeclampsia (PE). In the MBRN, GH was defined as systolic blood pressure ≥140 mm Hg and/or diastolic blood pressure ≥90 mm Hg after 20 weeks of gestation in a preconceptionally normotensive woman. PE additionally required proteinuria and/or other signs of significant end-organic dysfunction [26]. SGA was defined as a birth weight of less than 10^th^ percentile for gestational age and offspring sex. Preterm birth was defined as births before 37 weeks of gestation.

Given the limited number of placental abruption cases, pregnancies affected by this condition were included only within the composite adverse pregnancy outcome group rather than evaluated as a distinct exposure.

### Covariates

Covariates were age at HUNT4 participation, parity, highest obtained level of education, marital status, time since last birth, self-reported general health, smoking status, alcohol intake, and body mass index (BMI), calculated from standardized measurements of weight and height. We obtained information on maternal age, parity and time since birth from the MBRN. The remaining covariates were obtained from HUNT4.

### Statistical analyses

Descriptive characteristics of the study population are presented as means and standard deviations (SD) for continuous variables and as numbers and percentages for categorical variables. To compare differences between women with and without a history of APOs we used t-test for continuous variables and chi-squared-test for categorical variables.

For assessing associations between APOs and levels of MVPA respective total PA we used linear regression for continuous outcomes and logistic regression for categorical outcomes. Models were specified a priori based on previous literature and the relationships illustrated in the directed acyclic graph (DAG) (S1 Fig) Models were adjusted for a range of sociodemographic, health- and lifestyle-related covariates, including age (continuous, years), marital status (living with partner or not), education (professional certificate/high school or less, or greater than high school), parity (1, 2, 3 or ≥4), time since last birth (<1 year, 1 to <5 years, 5 to <10 years or ≥10 years), smoking status (never, former or current), alcohol intake (no alcohol consumption or alcohol consumption), BMI (underweight/normal weight (<25 kg/m^2^), overweight (25- < 30 kg/m^2^) or obese (≥30 kg/m^2^)), and self-reported health (poor/fair or good/excellent). In our initial model (Model 1), we adjusted for age at participation in HUNT4. In Model 2, we further adjusted for marital status, education, parity, and time since last birth. Model 3 was additionally adjusted for potentially modifiable covariates on postpartum lifestyle, including smoking status, alcohol intake, BMI, and self-reported general health. We regard model 2 as the main model to assess the association between APOs and PA. Model 3 was added to examine the potential contribution of other postpartum lifestyle factors and general health to this relationship. The results are presented as differences in MET-minutes/week and total PA min/day and 95% confidence intervals (CI) for linear regression. Women without APOs was the reference group. For logistic regression the results are presented as odds ratios (OR) and 95% CIs. Statistical analyses were performed using STATA/MP version 18.0 (StataCorp).

The data on MVPA and total PA follow a normal distribution. One potential outlier in MVPA MET-min/week was identified (6575.5 MET-min/week). The outlier was retained in the analysis to preserve the representativeness, and the inclusion did not affect the overall results.

### Sensitivity analyses

We conducted four additional sensitivity analyses. Chronic diseases can impact and restrict PA level. Therefore, we excluded women who had reported moderate or severe impairment due to physical illness or mental health problems in the questionnaire. Women can have different lifestyle patterns at different stages of life, particularly during early parenthood. We therefore restricted the analyses to women with 5 or less years since last birth. MBRN lacks information on previous births if the women moved to Norway after giving birth. These women could potentially have an APO in previous pregnancies. Thus, we analyzed the population excluding women with no information on previous births. Finally, we conducted analyses including women with unreliable measures of PA (<3 minutes of walking ≥1 day), to see how the exclusion of these women impacted the results. Since almost half of our initial population were excluded, mainly due to missing data on accelerometer measured PA, we also performed descriptive statistics of excluded and included population.

### Ethical approvals

All participants in HUNT4 have provided written informed consent for usage of de-identifiable health information in approved research projects, and for connecting their data to health registers. REK Midt-Norge approved the research project on December 7^th^, 2023 (reference no. 698119).

## Results

### Baseline characteristics

The present analysis included 3771 women, of whom 868 (23.0%) had a history of one or more APOs. Among the study population (N = 3771), 66 (1.8%) women were registered with GDM, 283 (7.5%) with HDP, 389 (10.3%) with SGA offspring, 304 (8.0%) with preterm birth and 23 (0.6%) women were registered with placental abruption before HUNT4 participation. A total of 703 women experienced one APO, while 165 women experienced two or more APOs (in the same or different pregnancies).

The characteristics of the study population are presented in Table 1. The mean age at HUNT4 participation was 40.7 years (SD 7.6), 67.7% of study participants had education beyond high school and 86.1% reported living with a partner. Slightly more than half of the participants were overweight or obese, 8.0% were current smokers and 78.2% reported any alcohol consumption. Among all, 13.5% of the women were primiparous, 41.7% had two, 33.0% had three and 11.8% had four or more births. A majority of participants (72.7%) had ≥ 5 years since their last birth, and 2.9% had given birth within a year before participating in HUNT4. Most of the participants (83.9%) reported good or excellent health. Compared to women with uncomplicated pregnancies, those with a history of APOs had higher parity, shorter time since last birth and lower education. Women with a history of APOs also had higher BMI and worse self-reported health.

**Table 1:** Descriptive characteristics of HUNT4 participants by history of adverse pregnancy outcomes.

	All participants (n = 3771)	Participants without APOs (n = 2903)	Participants with APOs (n = 868)	p-value*
Age, years	40.7 (7.6)	40.9 (7.7)	39.9 (7.1)	<0.01
Living with partner	3246 (86.1)	2487 (85.7)	759 (87.4)	0.19
Education				0.13
Professional certificate, high school or less	1220 (32.4)	921 (31.7)	299 (34.5)	
Greater than high school	2551 (67.7)	1982 (68.3)	569 (65.6)	
Parity				<0.01
1 birth	508 (13.5)	412 (14.2)	96 (11.1)	
2 births	1573 (41.7)	1195 (41.2)	378 (43.6)	
3 births	1244 (33.0)	980 (33.8)	264 (30.4)	
≥ 4 births	446 (11.8)	316 (10.9)	130 (15.0)	
Time since last birth, years				<0.01
< 1	109 (2.9)	81 (2.8)	28 (3.2)	
1 to <5	920 (24.4)	661 (22.8)	259 (29.8)	
5 to <10	991 (26.3)	735 (25.3)	256 (29.5)	
≥ 10	1751 (46.4)	1426 (49.1)	325 (37.4)	
General health				0.02
Poor/fair	609 (16.2)	447 (15.4)	162 (18.7)	
Good/excellent	3162 (83.9)	2456 (84.6)	706 (81.3)	
Smoking status				0.85
Never smoked	1966 (52.1)	1512 (52.1)	454 (52.3)	
Former smoker	1503 (39.9)	1162 (40.0)	341 (39.3)	
Current smoker	302 (8.0)	229 (7.9)	73 (8.4)	
Alcohol intake				0.02
No alcohol consumption	824 (21.9)	609 (21.0)	215 (24.8)	
Alcohol consumption	2947 (78.2)	2294 (79.0)	653 (75.2)	
BMI, kg/m^2^				<0.01
Underweight/normal weight (<25)	1746 (46.3)	1378 (47.5)	368 (42.4)	
Overweight (25–30)	1257 (33.3)	974 (33.6)	283 (32.6)	
Obese (≥30)	768 (20.4)	551 (19.0)	217 (25.0)	
Physical Activity (min/day)				
Slow walking	80.5 (24.4)	80.7 (24.6)	80.1 (23.8)	0.58
Moderate walking	15.3 (8.4)	15.5 (8.6)	14.8 (7.6)	0.03
Fast walking	11.2 (10.6)	11.4 (10.8)	10.2 (10.0)	<0.01
Running	1.6 (4.5)	1.7 (4.6)	1.5 (4.4)	0.41
Cycling	5.6 (6.9)	5.7 (6.7)	5.5 (7.6)	0.53
Total PA	114.3 (33.8)	115.0 (34.3)	112.1 (32.1)	0.03
Physical activity (MET-min/week)				
Slow walking	1578.5 (477.8)	1580.8 (481.5)	1570.7 (465.7)	0.58
Moderate walking	375.9 (205.1)	380.0 (210.3)	362.3 (186.1)	0.03
Fast walking	351.2 (335.4)	360.6 (340.6)	319.9 (315.2)	<0.01
Running	74.6 (206.7)	76.2 (209.2)	69.5 (198.1)	0.41
Cycling	228.6 (281.4)	230.2 (273.4)	223.4 (307.0)	0.53
MVPA, MET-min/week	1030.5 (610.6)	1047.0 (620.1)	975.1 (574.3)	<0.01
MVPA <500 MET-min/week	615 (16.3)	461 (15.9)	154 (17.7)	0.04

Values are presented as n (%) for categorical variables and mean (SD) for continuous variables.

APOs included are GDM (n = 66), HDP (n = 283), SGA (n = 389), placental abruption (n = 23) and preterm birth (n = 304).

Slow walking is defined as ≤4.0 km/h; moderate walking as 4.1–5.4 km/h; fast walking as ≥5.5 km/h. Total activity is defined as slow, moderate and fast walking, cycling and running.

*t-test for continuous variables/ chi-squared test for categorical variables.

Abbreviations: APOs, adverse pregnancy outcomes; GDM, gestational diabetes mellitus; HDP, hypertensive disorders of pregnancy including preeclampsia and gestational hypertension; MET, metabolic equivalent of task; MVPA, moderate-vigorous physical activity; PA, physical activity, SD, standard deviation; SGA, small for gestational age offspring.

The range of MVPA in the study population was 22.4–6575.5 MET-min/week, and the mean level was 1030.5 MET-min/week. Daily total PA had a range of 8.8–287.3 min/day and the mean level was 114.3 min/day.

### PA levels in women with and without APOs

Women with a history of APOs had less MVPA compared to women without APOs (975.1 (SD 574.3) MET-min/week vs. 1047.0 (SD 620.1) MET-min/week, p < 0.01), and less daily PA than women without APOs (112.1 (SD 32.1) min/day vs. 115.0 (SD 34.3) min/day, p = 0.03). Among women with uncomplicated pregnancies, 15.9% did not meet the recommended level of PA (<500 MET-min/week of MVPA), compared to 17.7% of the women with a history of APOs.

Univariable analysis found a negative association between APOs and both daily total PA and weekly MVPA. Further, covariates such as higher BMI and current smoking were associated with lower levels of weekly MVPA and daily PA. In contrast, self-reported good or excellent health was positively associated with both weekly MVPA and daily total PA. Compared with women who had one birth, those with higher parity had higher daily total PA. A time interval of more than one year since last birth was associated with higher levels of daily total PA. Weekly MVPA levels increased with longer time since childbirth (Table 2).

**Table 2:** Linear regression examining the associations between potential confounders and physical activity levels.

	MVPA (MET-min/week)		Total PA (min/day)	
	β-Coef.	95% CI	β-Coef.	95% CI
Univariable				
Adverse pregnancy outcome	−71.9	(−118.2, −25.7)	−2.9	(−5.4, −0.3)
Multivariable				
Adverse pregnancy outcome	−29.4	(−74.2, 15.5)	−1.8	(−4.3, 0.7)
Age, years	−3.2	(−7.2, 0.8)	0.4	(0.2, 0.6)
Living with a partner	24.8	(−31.0, 80.6)	6.1	(3.0, 9.2)
Education				
Low education	Ref		Ref	
High education	114.1	(72.0, 156.2)	−1.4	(−3.7, 1.0)
Parity				
1 birth	Ref		Ref	
2 births	14.4	(−45.9, 74.7)	5.3	(2.0, 8.7)
3 births	10.0	(−54.7, 74.6)	4.9	(1.3, 8.5)
>=4 births	42.8	(−37.7, 123.4)	6.7	(2.3, 11.2)
Time since last birth				
< 1	Ref		Ref	
1 to <5	135.1	(18.4, 251.9)	14.5	(8.0, 20.9)
5 to <10	262.7	(142.4, 383.0)	14.0	(7.3, 20.7)
>=10	377.5	(248.8, 506.2)	11.4	(4.3, 18.6)
Smoking				
Never	Ref		Ref	
Former	−33.5	(−73.9, 6.9)	−2.9	(−5.1, −0.6)
Current	−139.7	(−213.4, −66.1)	−3.0	(−7.1, 1.1)
Alcohol intake				
No alcohol consumption	Ref		Ref	
Alcohol consumption	43.8	(−2.6, 90.3)	−0.8	(−3.4, 1.8)
BMI				
Underweight/normal weight	Ref		Ref	
Overweight	−130.6	(−173.4, −87.9)	−5.6	(−7.95, −3.20)
Obese	−224.5	(−275.5, −173.6)	−14.9	(−17.8, −12.1)
Self-rated health				
Poor/fair	Ref		Ref	
Good/Excellent	213.6	(161.3, 266.0)	13.3	(10.4, 16.2)

Adverse pregnancy outcome includes history of gestational diabetes mellitus, hypertensive disorders of pregnancy, small for gestational age offspring, preterm birth and/or placental abruption in one or more pregnancies.

Univariable is unadjusted. Multivariable is adjusted for all included covariates.

Abbreviations: BMI, body mass index; CI, confidence interval; MET, metabolic equivalent of task; MVPA, moderate-vigorous physical activity; PA, physical activity, β-Coef, beta-coefficient.

**Fig 2:**
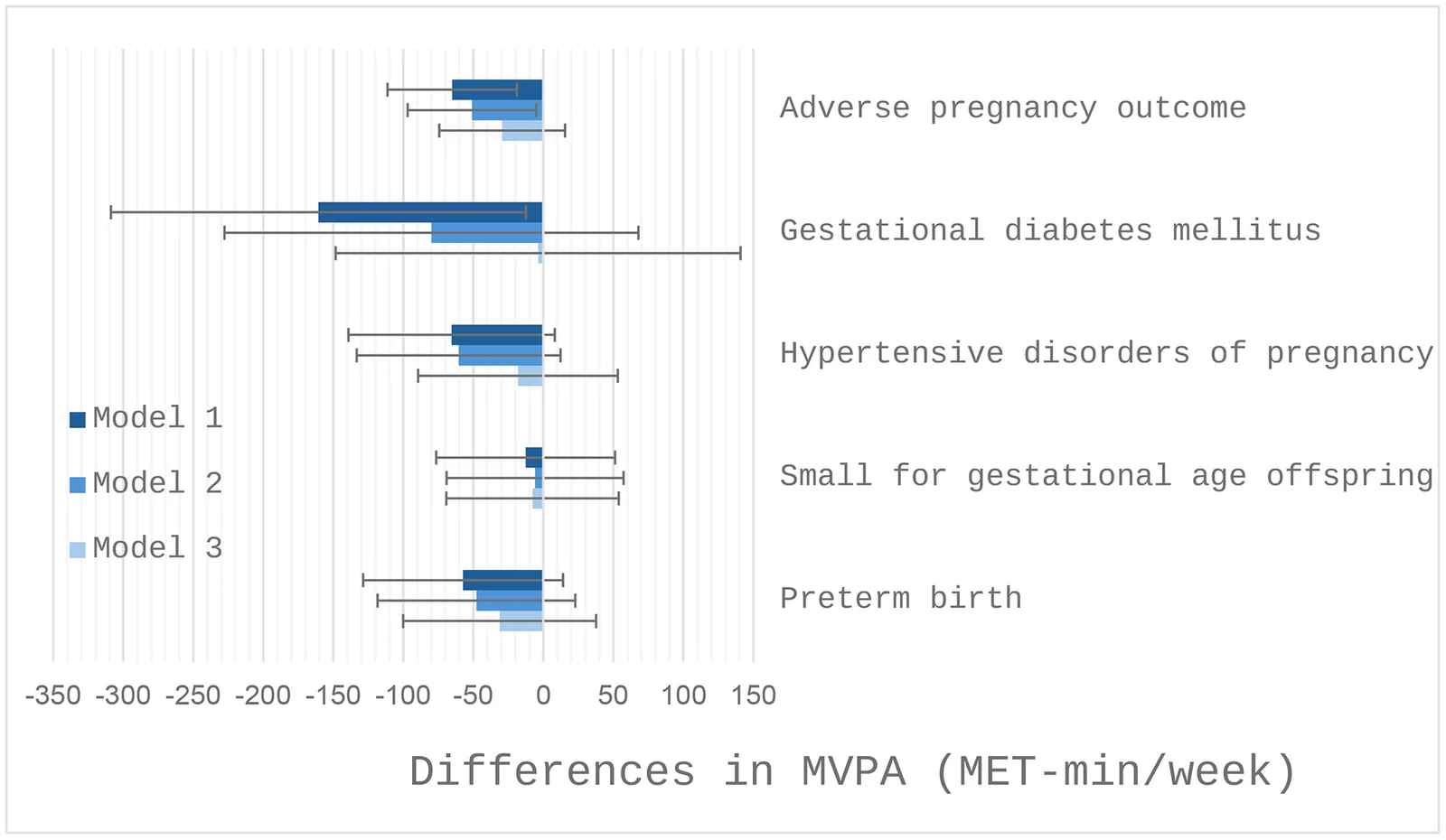
Associations between history of adverse pregnancy outcomes and moderate-to-vigorous physical activity (MVPA). Associations are estimated using linear regression; mean differences (95% CI) in MVPA for women with (N = 868) versus without (N = 2903) a history of adverse pregnancy outcomes. History of adverse pregnancy outcomes includes gestational diabetes mellitus, hypertensive disorders of pregnancy, small for gestational age offspring, preterm birth and/or placental abruption in one or more pregnancies. Model 1 is adjusted for age. Model 2 is adjusted for age, living with partner/no partner, education, parity and time since last birth. Model 3 is additionally adjusted for smoking, alcohol intake, BMI and self-reported general health.

**Fig 3:**
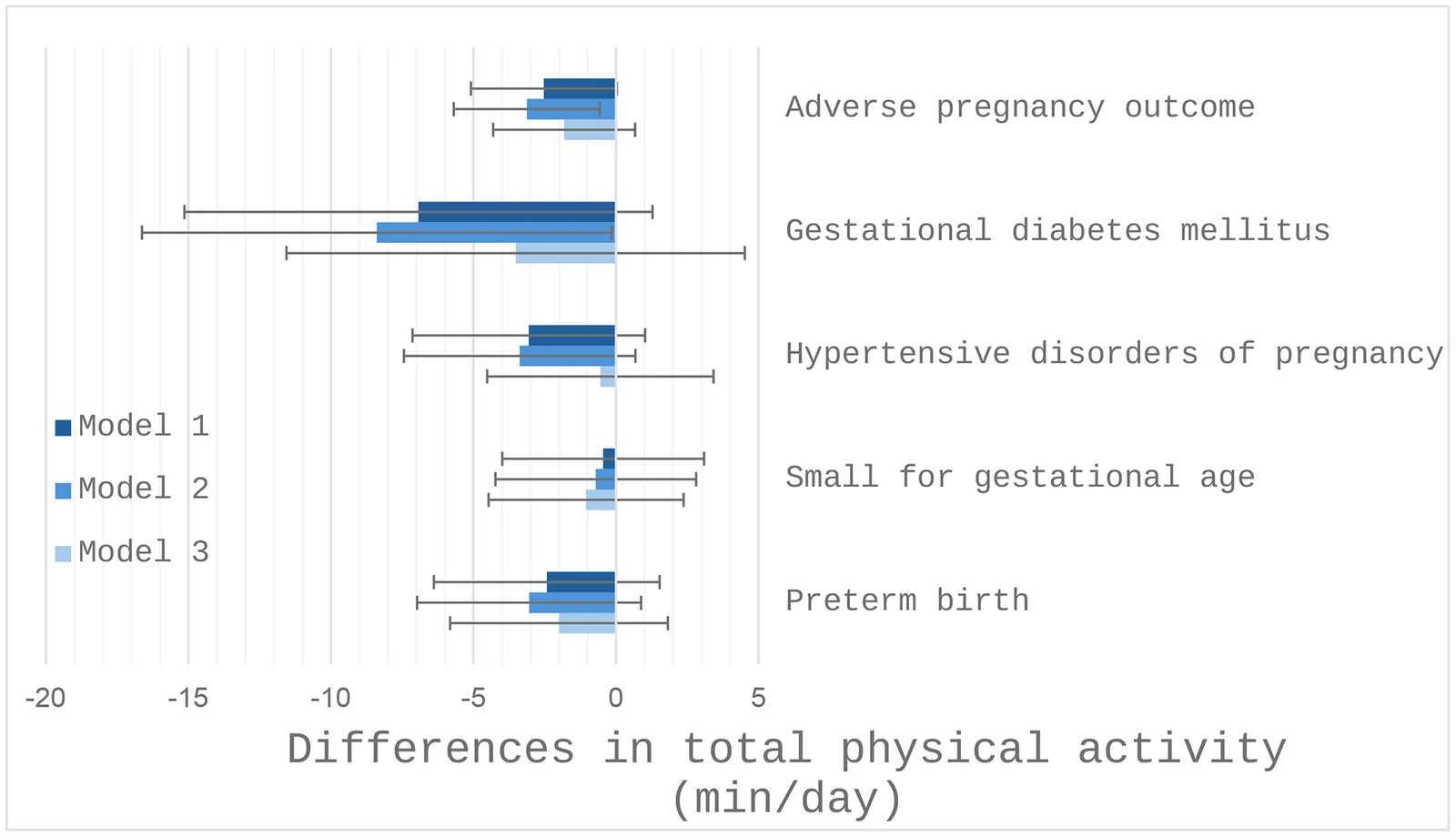
Associations between history of adverse pregnancy outcomes and total daily physical activity (min/day). Associations are estimated using linear regression; mean differences (95% CI) in PA min/day for women with (N = 868) versus without (N = 2903) a history of adverse pregnancy outcomes. History of adverse pregnancy outcomes includes gestational diabetes mellitus, hypertensive disorders of pregnancy, small for gestational age offspring, preterm birth and/or placental abruption in one or more pregnancies. Model 1 is adjusted for age. Model 2 is adjusted for age, living with partner/no partner, education, parity and time since last birth. Model 3 is additionally adjusted for smoking, alcohol intake, BMI and self-reported general health.

Adjusted linear regression analyses indicated that women with a history of APOs were generally less physically active than women without APOs (Figs 2 and 3, S1 Table). In models adjusted for age, marital status, education, parity, and time since last birth (Model 2), women with APOs engaged in less weekly MVPA (−51.0 MET-min/week (95% CI −96.9 to −5.1)) and less daily PA (−3.1 min/day (95% CI −5.7 to −0.6)) compared with women without APOs. After additional adjustment for post-pregnancy lifestyle factors and self-reported health (Model 3), these associations were attenuated and no longer statistically significant (MVPA: −29.4 MET-min/week; 95% CI −74.2 to 15.5; PA: −1.8 min/day; 95% CI −4.3 to 0.7). Separate analyses by APO subtype (GDM, HDP, SGA, preterm birth) suggested lower PA levels (both weekly MVPA and daily total PA) among affected women, but estimates were not statistically significant. Strongest association was seen in women with a history of GDM (Figs 2 and 3, S1 Table).

The age adjusted multivariate logistic regression model suggested that women with APOs may be less likely to achieve very high levels of weekly MVPA (>1000 MET-min/week), but this association was not present when adjusting for other covariates and lifestyle habits. There were no associations between different APOs and physical activity levels measured in MVPA or total daily PA. Odds ratios for being less or more physically active were close to 1, and confidence intervals consistently included the null (Table 3).

**Table 3:** Logistic regression analyses assessing the odds ratio of women with a history of adverse pregnancy outcomes (APOs) being less or more physical active than recommended (500-1000 MET-min/week) and having less or more total PA than the 33^rd^ to 66^th^ percentile compared to women without a history of APOs.

	Number of cases	Model 1*		Model 2*		Model 3*	
Adverse pregnancy outcomes	868						
MVPA (MET-min/week)		**OR**	**95% CI**	**OR**	**95% CI**	**OR**	**95% CI**
< 500	154	1.03	(0.83, 1.28)	1.01	(0.81, 1.25)	0.94	(0.75, 1.18)
500-1000	371	Ref		Ref		Ref	
> 1000	343	0.84	(0.71, 0.99)	0.87	(0.73, 1.03)	0.91	(0.77, 1.08)
Total PA (min/day)							
< 33 percentile	308	1.12	(0.93, 1.35)	1.15	(0.96, 1.39)	1.08	(0.89, 1.31)
33–66 percentile	282	Ref		Ref		Ref	
> 66 percentile	278	0.97	(0.80, 1.17)	0.94	(0.78, 1.14)	0.96	(0.79, 1.16)
Gestational diabetes mellitus	66						
MVPA (MET-min/week)		**OR**	**95% CI**	**OR**	**95% CI**	**OR**	**95% CI**
< 500	17	1.50	(0.82, 2.77)	1.27	(0.68, 2.38)	1.01	(0.53, 1.93)
500-1000	28	Ref		Ref		Ref	
> 1000	21	0.72	(0.41, 1.28)	0.85	(0.48, 1.51)	1.01	(0.56, 1.82)
Total PA (min/day)							
< 33 percentile	30	1.49	(0.84, 2.64)	1.63	(0.91, 2.92)	1.30	(0.72, 2.35)
33–66 percentile	20	Ref		Ref		Ref	
> 66 percentile	16	0.80	(0.41, 1.56)	0.76	(0.39, 1.48)	0.82	(0.42, 1.60)
Hypertensive disorders of pregnancy	283						
MVPA (MET-min/week)		**OR**	**95% CI**	**OR**	**95% CI**	**OR**	**95% CI**
< 500	45	0.87	(0.61, 1.25)	0.88	(0.62, 1.26)	0.76	(0.53, 1.09)
500-1000	126	Ref		Ref		Ref	
> 1000	112	0.82	(0.63, 1.07)	0.84	(0.64, 1.09)	0.92	(0.70, 1.21)
Total PA (min/day)							
< 33 percentile	105	1.13	(0.85, 1.52)	1.15	(0.86, 1.54)	1.01	(0.75, 1.36)
33–66 percentile	94	Ref		Ref		Ref	
> 66 percentile	84	0.87	(0.64, 1.18)	0.86	(0.63, 1.17)	0.90	(0.66, 1.23)
Small for gestational age offspring	389						
MVPA (MET-min/week)		**OR**	**95% CI**	**OR**	**95% CI**	**OR**	**95% CI**
< 500	66	0.96	(0.71, 1.30)	0.95	(0.70, 1.29)	0.97	(0.71, 1.33)
500-1000	169	Ref		Ref		Ref	
> 1000	154	0.84	(0.67, 1.06)	0.86	(0.68, 1.08)	0.86	(0.68, 1.09)
Total PA (min/day)							
< 33 percentile	125	0.93	(0.72, 1.21)	0.95	(0.73, 1.23)	0.96	(0.74, 1.25)
33–66 percentile	133	Ref		Ref		Ref	
> 66 percentile	131	0.97	(0.75, 1.25)	0.96	(0.75, 1.25)	0.95	(0.74, 1.23)
Preterm birth	304						
MVPA (MET-min/week)		**OR**	**95% CI**	**OR**	**95% CI**	**OR**	**95% CI**
< 500	49	0.91	(0.65, 1.28)	0.89	(0.63, 1.25)	0.83	(0.58, 1.18)
500-1000	132	Ref		Ref		Ref	
> 1000	123	0.87	(0.67, 1.12)	0.88	(0.68, 1.14)	0.92	(0.71, 1.20)
Total PA (min/day)							
< 33 percentile	113	1.16	(0.87, 1.54)	1.18	(0.89, 1.57)	1.13	(0.84, 1.51)
33–66 percentile	99	Ref		Ref		Ref	
> 66 percentile	92	0.91	(0.68, 1.22)	0.88	(0.66, 1.19)	0.88	(0.66, 1.19)

History of adverse pregnancy outcomes includes gestational diabetes mellitus, hypertensive disorders of pregnancy, small for gestational age, preterm birth and/or placental abruption in one or more pregnancies.

Model 1 is adjusted for age. Model 2 is adjusted for age, living with partner/no partner, education, parity and time since last birth. Model 3 is additionally adjusted for smoking, alcohol intake, BMI and self-reported general health.

Abbreviations: APOs, adverse pregnancy outcome; CI, confidence interval; MET, metabolic equivalent of task; MVPA, moderate-vigorous physical activity; PA, physical activity; OR, odds ratio.

### Sensitivity analyses

When excluding women reporting moderate or severe impairment due to physical illness or mental health problems, we observed greater differences in MVPA between women with and without a history of any APO (−51.2 MET-min/week, 95% CI, −98.3 to −4.2) in the fully adjusted model. Likewise, separate analyses of each APO with total PA (min/day) suggested greater differences in PA for all types of APOs compared to the main analysis, except for GDM. When we restricted our analyses to women with up to five years since last birth, we obtained similar results to the main analyses. Sensitivity analyses excluding women with missing information on first birth or including unreliable measures of PA yielded results comparable to the primary analyses (results not shown).

### Comparison of excluded and included population

A total of 4885 (56.4%) women were excluded from the study population, mainly because of missing data on device measured PA (n = 4177). S2 Table shows descriptive characteristics of the excluded and included population. Excluded women were slightly younger, had lower education, lower parity, and shorter time since last birth compared with included women. They also had higher BMI and were more likely to report poor or fair health (20.4% vs. 16.2%). Self-reported level of PA, categorized as active or inactive, was additionally compared. Self-reported frequency, intensity and duration of PA were combined to define inactivity as <150 minutes of PA per week [27]. Based on this definition, respectively 35.2% and 32.7% of the excluded and included women were classified as inactive.

## Discussion

The present study examined device-measured PA among women with and without a history of APOs. In unadjusted and partially adjusted models, a history of APOs was associated with lower levels of both total daily PA and weekly MVPA. However, these associations were attenuated and no longer statistically significant after adjustment for modifiable postpartum lifestyle factors and self-rated general health. Overall, 15.9% of women without APOs and 17.7% of women with a history of APOs did not achieve recommended levels of PA. Independent of APO status, lower PA was strongly associated with higher BMI, smoking and poor/fair self-reported general health, whereas higher levels of PA were associated with higher parity and longer time since last birth.

The current study is among the first to assess objectively measured PA levels in women after pregnancy. Previous research on the association between APOs and PA levels postpartum commonly used self-reported questionnaires on PA and generally found no differences or slightly lower PA levels in women with a history of APOs [14–20]. However, most studies consistently report low compliance with physical activity guidelines [15–17,20]. A recent Norwegian study using accelerometers among 182 women with and without history of HDP and/or GDM one year postpartum found that 14% of participants did not meet PA recommendations (≥150 min/week of moderate intensity or ≥75 min/week of vigorous intensity activity), with no significant differences between groups [13]. This aligns with our finding that 15.9–17.7% of women did not meet the recommendations for weekly moderate-to-vigorous physical activity (MVPA). Accelerometer data from the 2021–2022 Norwegian National Physical Activity Survey (NNPAS) found that approximately 22% of women of reproductive age (20–49 years) did not adhere to physical activity recommendations [28]. The higher compliance with PA recommendations in our study and in the study by Ziesler et al. may reflect that women participating in studies assessing PA tend to be more health-conscious and physically active [13].

In our analysis, women with a history of APOs tended to have slightly lower levels of daily total PA and weekly MVPA compared to women without APOs. The largest difference was found in MVPA, however, there was a wide range within groups. Even though most women in our study population (over 80%) reach the recommended levels of physical activity, the lower activity among women with a history of APOs remains a concern, as higher levels of activity beyond the minimum guidelines can provide substantial health benefits, including decreased CVD mortality [29]. The recommended PA levels aim to establish a minimum threshold of PA that are both achievable and safe for the general population [11]. While these guidelines are effective in promoting baseline health, they may underrepresent the full spectrum of benefits associated with higher levels of PA. A recent systematic review found that engaging in exercise first year postpartum reduced systolic and diastolic blood pressure as well as triglycerides [30]. To our knowledge, no studies have examined the dose–response relationship between PA and CVD risk reduction specifically in women with APOs.

Health literacy – the ability to access, understand, evaluate, and apply health information to make informed decisions about one’s health – is a key determinant of health behaviors. Higher health literacy is associated with adherence to healthy lifestyle recommendations as well as recognition of symptoms and timely health care seeking [31]. In our study, we found that educational level was associated with higher level of weekly MVPA. These findings reinforce the well-known association between level of education and lifestyle factors. A history of APOs represents a critical window of opportunity to improve long-term cardiovascular health. Clinical guidelines recommend follow-up preventive strategies for women after APOs to reduce future CVD risk [9,10]. However, evidence suggests that many women face low awareness of the link between APOs and future health risks. Additionally, gaps persist in clinician education, care coordination, and access to postpartum support services [32]. Addressing these barriers through targeted education, structured follow-up programs, and integrated care pathways could enhance risk awareness and promote healthier behaviors among women at increased risk.

Individual cardiovascular health can be assessed by four health behaviors (diet, physical activity, smoking, and sleep) and four metabolic factors (BMI, blood lipid, blood glucose, and blood pressure) [33]. A longitudinal study with 13 years follow-up found an inverse dose-response relationship between greater individual cardiovascular health and incident CVD in women with a history of APOs [33]. Importantly, among women with high cardiovascular health, cardiovascular risk was similar for those with and without APO history.

Although the current study links APO history to lower level of PA later in life, a longitudinal design would be ideal to track changes in PA over time and assess causality. PA engagement and intensity fluctuate across the life course, and the transition to parenthood is associated with a marked decline in PA level [34]. Further, prepregnancy PA is a strong predictor of PA in parenthood [35]. Evidence from two systematic reviews and meta-analysis have found an inverse association between physical activity prepregnancy and in early pregnancy and the risk of GDM [36] and PE [37]. These findings highlight the importance of preventive strategies aimed at increasing PA and optimizing lifestyle during the reproductive years, not only to reduce the likelihood of serious pregnancy complications but also to lower future CVD risk [3]. Our study opens avenues for exploring PA and exercise interventions and encouraging to PA postpartum, particularly for women at elevated CVD risk. Future research should also examine other lifestyle behaviors with cardiovascular impact, including diet, smoking and sleeping patterns.

Strengths of the current study include the large population-based design and linkage to comprehensive data from the MBRN. This gives the opportunity to adjust for a wide range of potential confounders, which improves the reliability of the findings and generalizability. Other strengths include the use of accelerometers, providing a valid and objective assessment of PA, compared to self-reported measures of PA in questionnaires. These questionnaires are prone to social desirability bias, leading to overestimation of high intensity PA and underestimation of sedentary behavior, making the results less reliable [21]. On the other side, behavior is likely to change when it is being monitored, regarding if the change is intended or not, a phenomenon called measurements reactivity [38]. However, MVPA seems to be less altered by reactivity [39]. Another strength is the broad inclusion of different APOs. There are limited studies that include preterm birth, SGA or placental abruption and their associations to PA levels, despite the well-known associations between these APOs and higher risk of CVD [15].

Limitations of the study include potential residual confounding from unmeasured or unknown factors which could influence PA levels. A proportion of eligible women were excluded, primarily due to missing device‑measured physical activity data. Excluded women were younger, had lower education, higher BMI, poorer self‑reported health, characteristics associated with lower physical activity. As a result, the analytic sample likely represents a healthier and more active subgroup, which may have attenuated differences between women with and without APOs and led to more conservative estimates of the total association. As PA was measured several years after pregnancy for most participants, our results reflect longer‑term activity rather than immediate postpartum behavior. Although this timing may attenuate group differences if the influence of APOs diminishes over time, both groups were assessed at similar postpartum intervals, making the internal comparison appropriate for our study aim. While the findings are robust within Norway, the generalizability to other populations may be limited. The prevalence of GDM in the study population is notably lower compared to international and newer Norwegian numbers, that estimates a prevalence of 5–7% [25]. This aligns with the observed increase of GDM diagnosis in MBRN over the past years [40]. In our study population we therefore may have a substantial number of undiagnosed GDM, especially among women who gave birth further back in time, leading to underestimation of the differences within the groups. Analyses of individual APOs may be affected by the limited number of cases for some subgroups, resulting in highly imprecise estimates and wide confidence intervals.

## Conclusion

In conclusion, we observed lower levels of device-measured MVPA among women with prior APOs relative to women with uncomplicated pregnancies, although these differences were attenuated after accounting for general health and modifiable lifestyle factors. Notably, nearly one of five woman (15.9–17.7%) did not meet recommended levels of weekly MVPA. The study highlights the intricate interplay between demographic characteristics, health status, lifestyle and PA levels. Intervention studies with the objective of increasing PA levels and studies that address which methods are more effective in increasing information about and motivation for a healthy lifestyle are warranted. Women with a history of APOs are at higher risk of CVD, and they may particularly benefit from increased PA levels. Future observational studies on lifestyle changes in women from pre- to postpartum are needed to further elucidate the relationship between APOs and physical activity trajectories.

## Supporting information

S1 Fig Directed acyclic graph (DAG) to illustrate the relationship between adverse pregnancy outcomes and physical activity. (PPTX)

S1 Table Linear regression analyses examining the associations between a history of APO and PA levels. (DOCX)

S2 Table Descriptive characteristics by inclusion status. (DOCX)
