# Supplementary material for: Device measured physical activity among women with and without adverse pregnancy outcomes: Data from the HUNT study
Source: PLoS One. 2026 Sep 8;21(9):e0357050. doi: 10.1371/journal.pone.0357050 (PMC13552725; doi:10.1371/journal.pone.0357050)
Supplement: Supplementary file 1 [file pone.0357050.s002.docx]

| **S1 Table. Linear regression analyses examining the associations between a history of APO and PA levels.** | | | | | |
| --- | --- | --- | --- | --- | --- |
|  | | **MVPA (MET-min/week)** | | **Total PA (min/day)** | |
| **Adverse pregnancy outcome** | | β-Coef. | 95% CI | β-Coef. | 95% CI |
|  | Model 1 | -65.1 | (-111.3, -18.9) | -2.5 | (-5.0, 0.0) |
|  | Model 2 | -51.0 | (-96.9, -5.1) | -3.1 | (-5.7, -0.6) |
|  | Model 3 | -29.4 | (-74.2, 15.5) | -1.8 | (-4.3, 0.7) |
|  |  |  |  |  |  |
| **Gestational diabetes mellitus** | | β-Coef. | 95% CI | β-Coef. | 95% CI |
|  | Model 1 | -160.6 | (-308.8, -12.4) | -6.9 | (-15.1, 1.3) |
|  | Model 2 | -79.9 | (-227.7, 67.8) | -8.4 | (-16.6, -0.2) |
|  | Model 3 | -3.7 | (-148.3, 141.0) | -3.5 | (-11.6, 4.5) |
|  |  |  |  |  |  |
| **Hypertensive disorders of pregnancy** | | β-Coef. | 95% CI | β-Coef. | 95% CI |
|  | Model 1 | -65.5 | (-139.2, 8.2) | -3.1 | (-7.2, 1.0) |
|  | Model 2 | -60.5 | (-133.3, 12.3) | -3.4 | (-7.4, 0.7) |
|  | Model 3 | -18.1 | (-89.4, 53.3) | -0.6 | (-4.5, 3.4) |
|  |  |  |  |  |  |
| **Small for gestational age offspring** | | β-Coef. | 95% CI | β-Coef. | 95% CI |
|  | Model 1 | -12.6 | (-76.5, 51.3) | -0.5 | (-4.0, 3.1) |
|  | Model 2 | -5.9 | (-69.1, 57.3) | -0.7 | (-4.2, 2.8) |
|  | Model 3 | -7.7 | (-69.3, 54.0) | -1.1 | (-4.5, 2.4) |
|  |  |  |  |  |  |
| **Preterm birth** | | β-Coef. | 95% CI | β-Coef. | 95% CI |
|  | Model 1 | -57.3 | (-128.7, 14.1) | -2.4 | (-6.4, 1.5) |
|  | Model 2 | -47.8 | (-118.4, 22.7) | -3.1 | (-7.0, 0.9) |
|  | Model 3 | -31.2 | (-100.1, 37.7) | -2.0 | (-5.8, 1.8) |
| Adverse pregnancy outcomes include history of gestational diabetes mellitus, hypertensive disorders of pregnancy, small for gestational age offspring, preterm birth and/or placental abruption in one or more pregnancies.  Model 1 was adjusted for age. Model 2 was adjusted for age, living with partner/no partner, education, parity and time since last birth. Model 3 was additionally adjusted for smoking, alcohol intake, BMI and self-reported general health.  Abbreviations: APO, adverse pregnancy outcome; PA, physical activity; MVPA, moderate-vigorous physical activity; MET, metabolic equivalent of task; β-Coef, β-coefficient; CI, confidence interval | | | | | |
