# Supplementary material for: Device measured physical activity among women with and without adverse pregnancy outcomes: Data from the HUNT study
Source: PLoS One. 2026 Sep 8;21(9):e0357050. doi: 10.1371/journal.pone.0357050 (PMC13552725; doi:10.1371/journal.pone.0357050)
Supplement: Supplementary file 2 [file pone.0357050.s003.docx]

| **S2 Table. Descriptive characteristics by inclusion status.** | | | | | | |
| --- | --- | --- | --- | --- | --- | --- |
|  | | | | **Excluded from the analyses (n=4885)** | **Included in the analyses (n=3771)** | **P-value*** |
| **Age, years, mean (SD)** | | | | 39.2 (8.1) | 40.7 (7.6) | <0.01 |
| **Living with partner** | | | | 4119 (84.3) | 3246 (86.1) | 0.02 |
| **Education** | | | |  |  | <0.01 |
|  | Professional certificate, high school or less | | | 2008 (41.1) | 1220 (32.4) |  |
|  | Greater than high school | | | 2834 (58.0) | 2551 (67.7) |  |
|  | Missing | | | 43 (0.9) | 0 (0) |  |
| **Parity** | | | |  |  | <0.01 |
|  | 1 birth | | | 930 (19.0) | 508 (13.5) |  |
|  | 2 births | | | 2038 (41.7) | 1573 (41.7) |  |
|  | 3 births | | | 1428 (29.2) | 1244 (33.0) |  |
|  | >=4 births | | | 489 (10.0) | 446 (11.8) |  |
| **Time since last birth, years** | | | |  |  | <0.01 |
|  | <1 | | | 143 (2.9) | 109 (2.9) |  |
|  | 1 to <5 | | | 1511 (30.9) | 920 (24.4) |  |
|  | 5 to <10 | | | 1203 (24.6) | 991 (26.3) |  |
|  | >=10 | | | 2028 (41.5) | 1751 (46.4) |  |
| **General health** | | | |  |  | <0.01 |
|  | Poor/fair | | | 994 (20.4) | 609 (16.2) |  |
|  | Good/excellent | | | 3784 (77.5) | 3,162 (83.9) |  |
|  | Missing | | | 107 (2.2) | 0 (0) |  |
| **Smoking status** | | | |  |  | <0.01 |
|  | Never smoked | | | 2411 (49.4) | 1966 (52.1) |  |
|  | Former smoker | | | 1941 (39.7) | 1503 (39.9) |  |
|  | Current smoker | | | 487 (10.0) | 302 (8.0) |  |
|  | Missing | | | 46 (0.9) | 0 (0) |  |
| **Alcohol intake** | | | |  |  | <0.01 |
|  | No alcohol consumption | | | 1049 (21.5) | 824 (21.9) |  |
|  | Alcohol consumption | | | 3064 (62.7) | 2947 (78.2) |  |
|  | Missing data | | | 772 (15.8) | 0 (0) |  |
| **BMI, kg/m^2^** | | | |  |  | <0.01 |
|  | Underweight/normal weight (<25) | | | 2003 (41.0) | 1746 (46.3) |  |
|  | Overweight (25-<30) | | | 1540 (31.5) | 1257 (33.3) |  |
|  | Obese (>=30) | | | 1092 (22.4) | 768 (20.4) |  |
|  | Missing | | | 250 (5.1) | 0 (0) |  |
| **Self-reported physical activity** | | | |  |  | <0.01 |
|  | | Inactive | | 1,721 (35.2) | 1233 (32.7) |  |
|  | | Active | | 2,192 (44.9) | 1964 (52.1) |  |
|  | | Missing | | 972 (19.9) | 574 (15.2) |  |
| **History of APO** | | | | 1,063 (21.8) | 868 (23.0) | 0.16 |
|  | | | GDM | 102 (2.1) | 66 (1.8) | 0.26 |
|  | | | HDP | 335 (6.9) | 283 (7.5) | 0.25 |
|  | | | SGA | 470 (9.6) | 389 (10.3) | 0.28 |
|  | | | Preterm birth | 375 (7.7) | 304 (8.1) | 0.51 |
|  | | | Placental abruption | 26 (0.5) | 23 (0.6) | 0.63 |
| Values are presented as n (%) for categorical variables and mean (SD) for continuous variables  *t-test for continuous variables/ chi-squared test for categorical variables  Abbreviations: APO, adverse pregnancy outcome; GDM, gestational diabetes mellitus; HDP, hypertensive disorders of pregnancy including preeclampsia and gestational hypertension; SD, standard deviation; SGA, small for gestational age offspring. | | | | | | |
