# Supplementary material for: Device measured physical activity among women with and without adverse pregnancy outcomes: Data from the HUNT study
Source: PLoS One. 2026 Sep 8;21(9):e0357050. doi: 10.1371/journal.pone.0357050 (PMC13552725; doi:10.1371/journal.pone.0357050)
Supplement: Supplementary file 3 [file pone.0357050.s004.docx]

PONE-D-26-01306

Device measured physical activity among women with and without adverse pregnancy outcomes: data from the HUNT study.

**Reviewers’ comments:**

**Reviewer #1**:

This manuscript examines device-measured physical activity (PA) among women with and without a history of adverse pregnancy outcomes (APOs) using linked data from the HUNT study and the Medical Birth Registry of Norway. The research question is clinically and public-health relevant, given the growing recognition of APOs as early markers of long-term cardiometabolic risk.

Overall, the study is clearly written. The use of accelerometer-based PA rather than self-reported measures is a major strength, as is the large population-based design and high-quality registry linkage.

Major comments:

**1.Explicit causal framework and DAG are required**
The manuscript currently lacks an explicit causal framework for the relationships among APOs, physical activity, and the covariates included in the models. The reason for adding those variables to each model is therefore unclear and hard to interpret. Without a directed acyclic graph (DAG) or equivalent causal justification, it is unclear whether these variables are intended to function as confounders, mediators, or both.
A DAG is essential to:
• clarify the causal estimand (e.g., total vs direct effect);
• justify the choice and ordering of covariate adjustment;
• prevent misinterpretation of the fully adjusted model as representing a “true” causal effect;
• avoid overadjustment and collider bias.
Recommendation:
I strongly recommend that the authors include a DAG (in the main text or supplementary material) illustrating:
• the temporal ordering of APOs, cardiometabolic health, lifestyle factors, and PA;
• which variables are considered confounders versus mediators;
• the rationale for the sequential modeling strategy.
If the intent of the analysis is descriptive rather than causal, this should also be explicitly stated. However, given the framing and interpretation of results, an explicit causal framework is necessary.

***Authors response:*** We thank the reviewer for this comment. We agree that an explicit causal framework improves clarity. We have now added a DAG in the Supplementary Material (S1 Fig.) outlining the assumed temporal ordering between adverse pregnancy outcomes (APOs), pre-pregnancy lifestyle factors, sociodemographic variables, postpartum lifestyle factors, and postpartum physical activity. The DAG clarifies which variables are conceptualized as confounders (e.g., age, education, marital status, parity, pre-pregnancy lifestyle factors) and which may lie on the causal pathway (e.g., postpartum body mass index (BMI), postpartum lifestyle factors). The study lacked data on pre‑pregnancy lifestyle factors, but we included them in the DAG to depict their theoretical relationship with post‑pregnancy lifestyle and adverse pregnancy outcomes. Based on the DAG, we now explicitly state that our primary objective is to estimate the total association between APOs and postpartum cardiometabolic health. We emphasize that the fully adjusted models are not intended to represent a direct causal effect but to illustrate attenuation patterns when adjusting for potential mediators.

This is now clarified in the Methods section:

Methods, page 10, line 204-206: “*We regard model 2 as the main model to assess the association between APOs and PA. Model 3 was added to examine the potential contribution of other postpartum lifestyle factors and general health to this relationship*.”

**2. Clarification of the causal estimand**
Although the study is described as a population-based cohort analysis, the causal estimand is not explicitly defined. It is therefore unclear whether the authors aim to estimate:
• the total effect of a history of APOs on later physical activity,
• a direct effect not operating through post-pregnancy health and lifestyle factors, or
• a purely descriptive association.

Please explicitly state the estimand of interest and frame the analysis and conclusions accordingly. Clarifying whether the goal is causal inference or descriptive epidemiology would substantially improve interpretability.

***Response:*** Thank you for this helpful suggestion. We have revised the aim of the study to more clearly reflect the estimand of interest. The updated wording clarifies that our objective is to compare differences in physical activity levels between women with and without a history of APOs (descriptive epidemiology), rather than to estimate a direct causal effect. This revision ensures that the aim is consistent with the causal structure presented in the DAG and aligns the framing of the study with our analytical approach. In the methods section we have added a sentence about the DAG and are referring to the model as supplementary material.

Introduction section, page 5, Line 77-83: “The primary aim of this study is to compare device-measured physical activity (PA) levels during the postpartum period between women with and without a history of adverse pregnancy outcomes (APOs). By employing accelerometry rather than self-report, the study addresses a critical gap in objective surveillance of PA in this high-risk population. Generating robust empirical evidence on PA patterns among women with prior APOs will inform the development of targeted postpartum interventions, with the overarching goal of improving maternal health and mitigating long-term risk.*”*

Methods section, page 9, line 192-193: *“Models were specified a priori based on previous literature and the relationships illustrated in the directed acyclic graph (DAG) (S1 fig.).”*

**3. Adjustment for post-exposure variables and overadjustment**
In the fully adjusted model (Model 3), the authors adjust for BMI, smoking, alcohol intake, and self-reported general health. These variables are measured at or near the time of PA assessment and plausibly lie downstream of APOs. As such, they may function as mediators rather than confounders.
From a causal perspective, adjusting for these factors likely blocks part of the effect pathway from APOs to later physical activity and may bias estimates toward the null. Therefore, attenuation of associations in Model 3 should not be interpreted as evidence that APOs are not associated with PA.

***Response:*** We agree with the reviewer that postpartum BMI, lifestyle factors, and self‑reported health may lie on the causal pathway between APOs and later physical activity. We have clarified that our primary estimand is the total association, and that Model 3 does not represent a direct causal effect but an exploratory attenuation model. As stated in the first comment, we regard model 2 as the main model to assess the association between APOs and PA. This is clarified in the methods (page 10, line 204-206).

**4. Interpretation of sequential models**
The stepwise adjustment strategy is clearly presented; however, the Results and Conclusions imply that the most adjusted model represents the “true” association. Without an explicit causal framework (e.g., a directed acyclic graph), this interpretation may be misleading.

***Response:*** We agree with the reviewer and have added a DAG to the Supplemental material (S1 Fig.).

**5. Selection bias due to missing accelerometer data**
More than 56% of eligible women were excluded, primarily due to missing device-measured PA data.
Excluded women were younger, less educated, had higher BMI, poorer self-rated health, and shorter time since last birth, characteristics strongly associated with physical activity. This suggests that the analytic sample may represent a healthier and more active subgroup, potentially attenuating differences between women with and without APOs. It's recommended to expand the discussion of potential selection bias and implications for generalizability.

***Response:*** Thank you for this important comment. We agree that the high proportion of excluded women, largely due to missing device‑measured physical activity data, may limit generalizability. As the noted, excluded women had lower education, higher BMI and poorer self-reported health. These characteristics are also associated with lower physical activity levels. Therefore, the analytic sample likely represents a somewhat healthier and more active subgroup of the eligible population. This is added in the revised manuscript.

Discussion section (limitations), page 27, line 425-430***:*** “*A proportion of eligible women were excluded, primarily due to missing device‑measured physical activity data. Excluded women were younger, had lower education, higher BMI, poorer self‑reported health, characteristics associated with lower physical activity. As a result, the analytic sample likely represents a healthier and more active subgroup, which may have attenuated differences between women with and without APOs and led to more conservative estimates of the total association”.*


**6. Timing and life-course interpretation**
Physical activity was measured many years after pregnancy for most participants, with nearly three-quarters having ≥5 years since their last birth. PA behaviors change substantially across the life course, and the influence of pregnancy-related complications may diminish over time. Clarify that the findings primarily reflect longer-term or mid-life physical activity, not immediate postpartum behavior, and discuss how this timing affects interpretation and implications for postpartum or early prevention interventions.

***Response:*** Thank you for this comment. We agree that physical activity changes across the life course and that measurement several years after pregnancy reflects longerterm activity rather than immediate postpartum behavior. However, because our aim is to compare PA levels between women with and without a history of APO, and because both groups were assessed at similar postpartum intervals, the timing does not affect the group comparison. We now explicitly note that while the influence of APOs may weaken over time, potentially attenuating associations, the internal comparison remains appropriate for addressing our study aim. In the revised manuscript we have clarified that in the discussion.

Discussion section (limitations), page 27, line 430-435***: “****As PA was measured several years after pregnancy for most participants, our results reflect longer‑term activity rather than immediate postpartum behavior. Although this timing may attenuate group differences if the influence of APOs diminishes over time, both groups were assessed at similar postpartum intervals, making the internal comparison appropriate for our study aim.”*

**Minor comments**
1. Consider noting that accelerometer-derived PA estimates are often higher than self-reported measures and may include incidental or occupational activity.

***Response:*** Thank you for this comment. We agree that accelerometers capture incidental and occupational activity that self‑reported measures may miss. However, it is well established that accelerometer derived PA estimates are generally lower than self‑reported PA, as questionnaires tend to overestimate activity due to recall and social desirability bias. Because our study relies exclusively on objective accelerometer data and does not compare device‑based measures with self‑report, we did not add this point to the manuscript, but we appreciate the opportunity to clarify this distinction in our response.

2. Use consistent terminology for “moderate-to-vigorous physical activity (MVPA)” throughout the manuscript.

***Respond:*** Thank you for the comment. We have reviewed the manuscript to ensure consistent use of terminology. “Physical activity (PA)” is used when referring to total activity including light-intensity activities (e.g., slow walking), whereas “moderate-to-vigorous physical activity (MVPA)” is used specifically for higher-intensity activity. These terms are now used consistently throughout the manuscript.

3. Given the very small number of placental abruption cases, explicitly state that estimates for this subgroup are highly uncertain.

***Respond:*** Thank you for this comment. We agree that the number of placental abruption cases was too low to allow reliable estimation of its association with postpartum physical activity. We agree that the number of placental abruption cases was too low to allow reliable estimation of its association with postpartum physical activity. Therefore, such cases were incorporated solely into the composite adverse pregnancy outcome group rather than evaluated as an independent exposure. This is now clarified in the manuscript.

Methods section, page 9, line175-177: *“Given the limited number of placental abruption cases, pregnancies affected by this condition were included only within the composite adverse pregnancy outcome group rather than evaluated as a distinct exposure.”*

4. Adding sample sizes to Figures 2 and 3 would improve readability and interpretation.

***Respond:*** Thank you for the comment. We have added number of women with and without APOs in the figure legend.

**Reviewer #2**:

Suggest to include more explanation in result section for the abstract
for line 59-60 (add more references)
for line 343 include reference for Ziesler et al
Good methodology and discussion, robust study

***Response:*** Thank you for your feedback. In the revised manuscript we have included more references and included Ziesler et al when sited.
