# Supplementary material for: Device measured physical activity among women with and without adverse pregnancy outcomes: Data from the HUNT study
Source: PLoS One. 2026 Sep 8;21(9):e0357050. doi: 10.1371/journal.pone.0357050 (PMC13552725; doi:10.1371/journal.pone.0357050)
Supplement: Peer review history

# Peer review: Decision Letter 0

**DOI:** 10.1371/journal.pone.0357050.r001

**Authors:** Dan Shan

24 Feb 2026

Dear Dr. Stafne,

Thank you for submitting your manuscript to PLOS ONE. After careful consideration, we feel that it has merit but does not fully meet PLOS ONE’s publication criteria as it currently stands. Therefore, we invite you to submit a revised version of the manuscript that addresses the points raised during the review process.

The manuscript has been evaluated by two external reviewers. Both reviewers consider the study timely and potentially valuable, but they have also raised a number of points that must be addressed before the manuscript can be considered further. Therefore, I invite you to submit a revised version (Major Revision) that responds fully to the reviewers’ comments.

Required revision components

Please prepare and upload the following items with your resubmission:

1. Point-by-point response to reviewers

Provide a detailed, numbered response to every comment from Reviewer 1 and Reviewer 2.

Upload a clean revised manuscript.

Upload a tracked-changes/marked-up version (or otherwise clearly highlight changes).

3.STROBE checklist (required)

As this is an observational study, please upload a completed STROBE checklist.

Indicate the manuscript page/line numbers where each STROBE item is addressed.

If any items are not applicable, please state this clearly in the checklist.

4.Additional reminders (PLOS ONE reporting expectations)

As relevant to the reviewer comments, please ensure the revision clearly reports: cohort selection and registry linkage, APO definitions and classification, device-based physical activity processing (wear-time, valid days, thresholds/cut-points), missing data handling, statistical modeling and covariate rationale (including consideration of postpartum health and modifiable lifestyle factors), and robustness/sensitivity analyses where appropriate. Please also confirm compliance with PLOS ONE policies on ethics and data availability.

Please submit your revised manuscript by Apr 10 2026 11:59PM. If you will need more time than this to complete your revisions, please reply to this message or contact the journal office at plosone@plos.org

If you would like to make changes to your financial disclosure, please include your updated statement in your cover letter. Guidelines for resubmitting your figure files are available below the reviewer comments at the end of this letter.

If applicable, we recommend that you deposit your laboratory protocols in protocols.io to enhance the reproducibility of your results. Protocols.io assigns your protocol its own identifier (DOI) so that it can be cited independently in the future. For instructions see: https://journals.plos.org/plosone/s/submission-guidelines#loc-laboratory-protocols. Additionally, PLOS ONE offers an option for publishing peer-reviewed Lab Protocol articles, which describe protocols hosted on protocols.io. Read more information on sharing protocols at https://plos.org/protocols?utm_medium=editorial-email&utm_source=authorletters&utm_campaign=protocols.

We look forward to receiving your revised manuscript.

Kind regards,

Dan Shan

Academic Editor

PLOS One

Journal requirements:

When submitting your revision, we need you to address these additional requirements.

1. Please ensure that your manuscript meets PLOS ONE's style requirements, including those for file naming. The PLOS ONE style templates can be found at

https://journals.plos.org/plosone/s/file?id=wjVg/PLOSOne_formatting_sample_main_body.pdf and

https://journals.plos.org/plosone/s/file?id=ba62/PLOSOne_formatting_sample_title_authors_affiliations.pdf

2. We note that you have indicated that there are restrictions to data sharing for this study. For studies involving human research participant data or other sensitive data, we encourage authors to share de-identified or anonymized data. However, when data cannot be publicly shared for ethical reasons, we allow authors to make their data sets available upon request. For information on unacceptable data access restrictions, please see http://journals.plos.org/plosone/s/data-availability#loc-unacceptable-data-access-restrictions.

Before we proceed with your manuscript, please address the following prompts:

a) If there are ethical or legal restrictions on sharing a de-identified data set, please explain them in detail (e.g., data contain potentially identifying or sensitive patient information, data are owned by a third-party organization, etc.) and who has imposed them (e.g., a Research Ethics Committee or Institutional Review Board, etc.). Please also provide contact information for a data access committee, ethics committee, or other institutional body to which data requests may be sent.

b) If there are no restrictions, please upload the minimal anonymized data set necessary to replicate your study findings to a stable, public repository and provide us with the relevant URLs, DOIs, or accession numbers. Please see http://www.bmj.com/content/340/bmj.c181.long for guidelines on how to de-identify and prepare clinical data for publication. For a list of recommended repositories, please see https://journals.plos.org/plosone/s/recommended-repositories. You also have the option of uploading the data as Supporting Information files, but we would recommend depositing data directly to a data repository if possible.

Please update your Data Availability statement in the submission form accordingly.

If the reviewer comments include a recommendation to cite specific previously published works, please review and evaluate these publications to determine whether they are relevant and should be cited. There is no requirement to cite these works unless the editor has indicated otherwise.

[Note: HTML markup is below. Please do not edit.]

Reviewers' comments:

Reviewer's Responses to Questions

**Comments to the Author**

1. Is the manuscript technically sound, and do the data support the conclusions?

Reviewer #1: Yes

Reviewer #2: Yes

**********

2. Has the statistical analysis been performed appropriately and rigorously?

Reviewer #1: Yes

Reviewer #2: Yes

**********

3. Have the authors made all data underlying the findings in their manuscript fully available?

The PLOS Data policy

Reviewer #1: No

Reviewer #2: Yes

**********

4. Is the manuscript presented in an intelligible fashion and written in standard English?

Reviewer #1: Yes

Reviewer #2: Yes

**********

Reviewer #1: This manuscript examines device-measured physical activity (PA) among women with and without a history of adverse pregnancy outcomes (APOs) using linked data from the HUNT study and the Medical Birth Registry of Norway. The research question is clinically and public-health relevant, given the growing recognition of APOs as early markers of long-term cardiometabolic risk.

Overall, the study is clearly written. The use of accelerometer-based PA rather than self-reported measures is a major strength, as is the large population-based design and high-quality registry linkage.

Major comments:

1.Explicit causal framework and DAG are required

The manuscript currently lacks an explicit causal framework for the relationships among APOs, physical activity, and the covariates included in the models. The reason for adding those variables to each model is therefore unclear and hard to interpret. Without a directed acyclic graph (DAG) or equivalent causal justification, it is unclear whether these variables are intended to function as confounders, mediators, or both.

A DAG is essential to:

• clarify the causal estimand (e.g., total vs direct effect);

• justify the choice and ordering of covariate adjustment;

• prevent misinterpretation of the fully adjusted model as representing a “true” causal effect;

• avoid overadjustment and collider bias.

Recommendation:

I strongly recommend that the authors include a DAG (in the main text or supplementary material) illustrating:

• the temporal ordering of APOs, cardiometabolic health, lifestyle factors, and PA;

• which variables are considered confounders versus mediators;

• the rationale for the sequential modeling strategy.

If the intent of the analysis is descriptive rather than causal, this should also be explicitly stated. However, given the framing and interpretation of results, an explicit causal framework is necessary.

2. Clarification of the causal estimand

Although the study is described as a population-based cohort analysis, the causal estimand is not explicitly defined. It is therefore unclear whether the authors aim to estimate:

• the total effect of a history of APOs on later physical activity,

• a direct effect not operating through post-pregnancy health and lifestyle factors, or

• a purely descriptive association.

Please explicitly state the estimand of interest and frame the analysis and conclusions accordingly. Clarifying whether the goal is causal inference or descriptive epidemiology would substantially improve interpretability.

3. Adjustment for post-exposure variables and overadjustment

In the fully adjusted model (Model 3), the authors adjust for BMI, smoking, alcohol intake, and self-reported general health. These variables are measured at or near the time of PA assessment and plausibly lie downstream of APOs. As such, they may function as mediators rather than confounders.

From a causal perspective, adjusting for these factors likely blocks part of the effect pathway from APOs to later physical activity and may bias estimates toward the null. Therefore, attenuation of associations in Model 3 should not be interpreted as evidence that APOs are not associated with PA.

4. Interpretation of sequential models

The stepwise adjustment strategy is clearly presented; however, the Results and Conclusions imply that the most adjusted model represents the “true” association. Without an explicit causal framework (e.g., a directed acyclic graph), this interpretation may be misleading.

5. Selection bias due to missing accelerometer data

More than 56% of eligible women were excluded, primarily due to missing device-measured PA data.

Excluded women were younger, less educated, had higher BMI, poorer self-rated health, and shorter time since last birth, characteristics strongly associated with physical activity. This suggests that the analytic sample may represent a healthier and more active subgroup, potentially attenuating differences between women with and without APOs. It's recommended to expand the discussion of potential selection bias and implications for generalizability.

6. Timing and life-course interpretation

Physical activity was measured many years after pregnancy for most participants, with nearly three-quarters having ≥5 years since their last birth. PA behaviors change substantially across the life course, and the influence of pregnancy-related complications may diminish over time. Clarify that the findings primarily reflect longer-term or mid-life physical activity, not immediate postpartum behavior, and discuss how this timing affects interpretation and implications for postpartum or early prevention interventions.

Minor comments

1. Consider noting that accelerometer-derived PA estimates are often higher than self-reported measures and may include incidental or occupational activity.

2. Use consistent terminology for “moderate-to-vigorous physical activity (MVPA)” throughout the manuscript.

3. Given the very small number of placental abruption cases, explicitly state that estimates for this subgroup are highly uncertain.

4. Adding sample sizes to Figures 2 and 3 would improve readability and interpretation.

Reviewer #2: suggest to include more explanation in result section for the abstract

for line 59-60 (add more references)

for line 343 include reference for Ziesler et al

Good methodology and discussion, robust study

**********

what does this mean?). If published, this will include your full peer review and any attached files.

If you choose “no”, your identity will remain anonymous but your review may still be made public.

**Do you want your identity to be public for this peer review?**  For information about this choice, including consent withdrawal, please see our Privacy Policy

Reviewer #1: No

Reviewer #2: No

**********

[NOTE: If reviewer comments were submitted as an attachment file, they will be attached to this email and accessible via the submission site. Please log into your account, locate the manuscript record, and check for the action link "View Attachments". If this link does not appear, there are no attachment files.]

To ensure your figures meet our technical requirements, please review our figure guidelines: https://journals.plos.org/plosone/s/figures

You may also use PLOS’s free figure tool, NAAS, to help you prepare publication quality figures: https://journals.plos.org/plosone/s/figures#loc-tools-for-figure-preparation.

NAAS will assess whether your figures meet our technical requirements by comparing each figure against our figure specifications.

# Author comment: Author response to Decision Letter 1

**DOI:** 10.1371/journal.pone.0357050.r002

11 Apr 2026

Please find uploaded the Point-by-point response to reviewers. revised manuscript (clean version and with tracked-changes) and STROBE checklist. In the coverletter we have explained why data sharing is not possible.

Thank you for the ability to revise the manuscript.

Attachment Submitted filename: PLOSONE_response to reviewer.docx

# Editor report: Decision Letter 1

**DOI:** 10.1371/journal.pone.0357050.r003

**Authors:** Francesca Crovetto

11 Aug 2026

Device measured physical activity among women with and without adverse pregnancy outcomes: data from the HUNT study

PONE-D-26-01306R1

Dear Dr. Signe Nilssen

We’re pleased to inform you that your manuscript has been judged scientifically suitable for publication and will be formally accepted for publication once it meets all outstanding technical requirements.

Within one week, you’ll receive an e-mail detailing the required amendments. When these have been addressed, you’ll receive a formal acceptance letter and your manuscript will be scheduled for publication.

An invoice will be generated when your article is formally accepted. Please note, if your institution has a publishing partnership with PLOS and your article meets the relevant criteria, all or part of your publication costs will be covered. Please make sure your user information is up-to-date by logging into Editorial Manager at Editorial Manager® and clicking the ‘Update My Information' link at the top of the page. For questions related to billing, please contact billing support.

If your institution or institutions have a press office, please notify them about your upcoming paper to help maximize its impact. If they’ll be preparing press materials, please inform our press team as soon as possible -- no later than 48 hours after receiving the formal acceptance. Your manuscript will remain under strict press embargo until 2 pm Eastern Time on the date of publication. For more information, please contact onepress@plos.org.

Kind regards,

Francesca Crovetto

Academic Editor

PLOS One

# Editor report: Acceptance letter

**DOI:** 10.1371/journal.pone.0357050.r004

**Authors:** Francesca Crovetto

PONE-D-26-01306R1

PLOS One

Dear Dr. Stafne,

I'm pleased to inform you that your manuscript has been deemed suitable for publication in PLOS One. Congratulations! Your manuscript is now being handed over to our production team.

At this stage, our production department will prepare your paper for publication. This includes ensuring the following:

* All references, tables, and figures are properly cited

* All relevant supporting information is included in the manuscript submission,

* There are no issues that prevent the paper from being properly typeset

You will receive further instructions from the production team, including instructions on how to review your proof when it is ready. Please keep in mind that we are working through a large volume of accepted articles, so please give us a few days to review your paper and let you know the next and final steps.

Lastly, if your institution or institutions have a press office, please let them know about your upcoming paper now to help maximize its impact. If they'll be preparing press materials, please inform our press team within the next 48 hours. Your manuscript will remain under strict press embargo until 2 pm Eastern Time on the date of publication. For more information, please contact onepress@plos.org.

You will receive an invoice from PLOS for your publication fee after your manuscript has reached the completed accept phase. If you receive an email requesting payment before acceptance or for any other service, this may be a phishing scheme. Learn how to identify phishing emails and protect your accounts at https://explore.plos.org/phishing.

If we can help with anything else, please email us at customercare@plos.org.

Thank you for submitting your work to PLOS One and supporting open access.

Kind regards,

PLOS One Editorial Office Staff

on behalf of

Dr. Francesca Crovetto

Academic Editor

PLOS One
